# Identification and genetic characterization of *Toxoplasma gondii* in free-ranging bristle-spined porcupine (*Chaetomys subspinosus*), a threatened arboreal mammal from the Brazilian Atlantic Forest

**DOI:** 10.1186/s13071-015-0882-6

**Published:** 2015-05-17

**Authors:** Rodrigo Alves Bezerra, Gastón Andrés Fernandez Giné, Bianca Mendes Maciel, Fernanda Amato Gaiotto, George Rêgo Albuquerque

**Affiliations:** Departamento de Ciências Biológicas, Universidade Estadual de Santa Cruz – UESC, Rodovia Jorge Amado, Km 16, Salobrinho, Ilhéus, BA 45662-900 Brazil; Departamento de Ciências Agrárias e Ambientais, Universidade Estadual de Santa Cruz – UESC, Rodovia Jorge Amado, Km 16, Salobrinho, Ilhéus, BA 45662-900 Brazil

**Keywords:** Genetic diversity, Population structure, Genotype, Sequencing, Toxoplasmosis

## Abstract

**Background:**

Strains of *Toxoplasma gondii* in Brazil have high genetic diversity compared to North America and Europe. The bristle-spined porcupine, *Chaetomys subspinosus*, is often subject to hunting for human food, but it is not known whether it can be a reservoir of this parasite. The aim of this study was to verify the occurrence of *T. gondii* in *C. subspinosus* from southern Bahia, Brazil, and genetically characterize and compare the strains found with those isolated in previous studies of the same region to quantify their genetic diversity by multilocus PCR-RFLP and PCR sequencing.

**Findings:**

Twelve free-ranging *C. subspinosus* captured in forest fragments of the Una Biological Reserve and adjacent areas were evaluated. Three isolates of *T. gondii* (TgCsBr01-03) were detected. Two different genotypes were identified by applying multilocus PCR-RFLP with six molecular markers (SAG1, SAG2, SAG3, c22-8, PK1, and Apico). The isolates TgCsBr02 and TgCsBr03 were indistinguishable by this technique. However, the three isolates differed from all the reference strains and from the samples from the same region. Nevertheless, when the six genetic markers were used in multilocus PCR sequencing, all three isolates of *T. gondii* were different. The phylogenetic analysis revealed a greater genetic distance for TgCsBr01, which was closer to isolates from pigs from the same region, while TgCsBr02-03 was classified in the same lineage and was closer to isolates from sheep from this region.

**Conclusions:**

All the isolates differed from the clonal genotypes of types I, II, and III using both genotyping techniques.

## Background

*Toxoplasma gondii* is an obligate intracellular parasite that infects humans and a variety of warm-blooded animals as the intermediate hosts, with felines as the definitive hosts. *T. gondii* infections are widely prevalent in humans and animals throughout the world [1].

Molecular studies using PCR-RFLP and microsatellite analysis on isolates of *T. gondii* in North Africa, Europe, and North America classified the genetic lineages into three types, designated types I, II, III. However, the use of new molecular markers and the study of isolates from South America, especially Brazil, have shown that *T. gondii* has higher genetic variability [2–4].

Usually, wild animals are reservoirs of *T. gondii*, and the consumption of their raw or undercooked meat by humans may transport this protozoan [5, 6]. In the Neotropics, consumption of bushmeat from hunting activity by local people is common [7]. In addition to increasing the risk of human contamination by zoonoses, hunting in this biome subjects several species of mammals to direct risk of extinction [8]. This is the case of the bristle-spined porcupine, *Chaetomys subspinosus*, a rodent species (of the family Erethizontidae) that is an arboreal folivore [9] of medium size [7] and endemic to the Atlantic Forest, whose populations are subject to strong hunting pressure throughout its distribution area, mainly for human consumption [10].

The aim of this study was to verify the occurrence of *T. gondii* in *C. subspinosu*s from southern Bahia, northeastern Brazil, as well as to genetically characterize and compare the strains found with those isolated in previous studies of the same region to verify their genetic diversity through multilocus PCR-RFLP and PCR sequencing techniques.

## Findings

### Methods

#### Collection of samples of biological material

##### Ethical approval

Experimental samples (brain and blood) [11] were collected from 12 free-ranging adult bristle-spined porcupines from the Una Biological Reserve and adjacent areas, located in southern Bahia, Brazil, from January to November 2013. Of these 12 animals, 8 were captured, sedated [9], the other 4 were found dead by researchers or local residents. All the procedures were performed under the legal approval and consent of the Brazilian Federal Authority (ICMBio, license number: 25184–1; 23468–2 and 27021–1). The proposed study was approved by the ethics committee (CEUA-UESC 024/13).

#### Molecular diagnosis and genetic characterization of *Toxoplasma gondii*

##### DNA extraction

DNA from blood (8 animals) and brain (4 animals) samples was extracted using the commercial kit Easy-DNA™ (Invitrogen). Tachyzoites of the RH strain were diluted at (10^7^/mL) and homogenized, for use as a positive control. DNA was extracted and performed according to Bezerra *et al.* [3]. Samples stored at −20 °C.

##### Diagnosis by PCR

*T. gondii* was detected by polymerase chain reaction (PCR) which amplified a fragment of 529 bp utilizing the *primers* Tox4 Forward (CGCTGCAGGGAGGAAGACGAAAGTTG) and Tox5 reverse (CGCTGCAGACACAGTGCATCTGGATT) [12].

##### PCR-RFLP for genetic characterization

The genotypes of *T. gondii* isolated were determined by means of multilocus PCR-RFLP with six genetic markers: SAG1, SAG2, SAG3, c22-8, PK1, and Apico. The amplification reactions were performed according to Bezerra *et al.* [3]. The digestions were carried out according to Su *et al.* [13]. The patterns of the DNA bands of the samples were compared with the genotypes deposited in ToxoDB (http://toxodb.org/toxo/).

##### DNA sequencing

The products from nested PCR were purified using PureLink™ (Invitrogen) and sequenced for six genetic markers (SAG1, SAG2, SAG3, c22-8, PK1, and Apico) using the automatic sequencer *ABI-PRISM* 3100 *Genetic Analyzer* (Applied Biosystems). As positive controls, nested PCR products of RH (type I), PTG (type II), and CTG strains (type III) were sequenced. Nucleotide sequences determined in this study were assembled in contigs using CAP3. The sequences of *T. gondii* were aligned with ClustalW (version 1.83; [14]), manually corrected using BioEdit Sequence Alignment Editor, and compared with 10 reference sequences of *T. gondii* available at NCBI (http://www.ncbi.nlm.nih.gov/bioproject/). These strains were GT1 (PRJNA16727), ME49 (PRJNA28893), VEG (PRJNA19097), FOU (PRJNA61561), MAS (PRJNA61545), VAND (PRJNA60839), RUB (PRJNA61119), p89 (PRJNA61547), TgCATBr5 (PRJNA61551), and TgCATBr9 (PRJNA61549). For the Apico marker, the sequences were aligned with the *T. gondii* apicoplast complete genome (U87145.2). All the sequences were compared with sequences available at ToxoDB.

##### Phylogenetic and statistical analysis

The phylogenetic analysis was performed using MEGA version 6 by means of the neighbor-joining algorithm, and the distances were computed using the Tajima-Nei method. The stability of branches was assessed after bootstrapping with 500 replicates. To verify the distance between samples of the same geographical origin, eight *T. gondii* isolated from pigs from southern Bahia (TgPgBr06, TgPgBr08, TgPgBr09, TgPgBr11, TgPgBr12, TgPgBr13, TgPgBr15, and TgPgBr16) [3] and three *T. gondii* isolated from sheep from southern Bahia (#54, #124, and #127) [4] were included. Tajima’s test of neutrality [15] was used to compare the number of segregating sites with the nucleotide diversity of the DNA sequences.

## Results

### Genetic characterization by multilocus PCR-RFLP

Three (25 %) animals were positive for *T. gondii* based on the biological samples. The positive samples were from three different and adjacent forest fragments within the Una Biological Reserve: TgCsBr01, TgCsBr02 and TgCsBr03. The greatest distance between the positive records was approximately 6 km. The application of PCR-RFLP with six genetic markers (SAG1, SAG2, SAG3, c22-8, PK1, and Apico) revealed two genotype groups in the three isolates (Table 1).

**Table 1:** Multi-locus genotypes of Brazilian Toxoplasma gondii isolates by PCR-RFLP

	Genetic markers						
*T. gondii* isolates	SAG1	SAG2	SAG3	c22-8	PK1	Apico	Reference
From *C. subspinosus* in Brazil							
TgCsBr 01	I	I	III	I	III	III	This study
TgCsBr 02	I	I	III	III	I	III	
TgCsBr 03	I	I	III	III	I	III	
From reference strains (clonal types I, II and III)							
RH88 (I)	I	I	I	I	I	I	Dubey et al. [16]
CTg (II)	II/III	II	II	II	II	II	
PTg (III)	II/III	III	III	III	III	III	
From Brazilian genotypes							
BrI	I	I	III	u-1	I	I	Pena et al. [2] Dubey et al. [16]
BrII	I	II	III	I	II	III	
BrIII	I	III	III	II	III	III	
BrIV	u-1	I	III	u-1	III	I	
From the same geographic origin of this study – sheep							
#54	I	I	I	III	u-1	III	Maciel et al. [4]
#124	I	I	I	III	u-1	III	
#127	I	I	I	III	u-1	III	
From the same geographic origin of this study – pigs							
TgPgBr06/TgPgBr08/TgPgBr11/TgPgBr12/TgPgBr14/TgPgBr15	I	I	III	I	I	III	Bezerra et al. [3]
TgPgBr 7	I	I	III	u-1	ND	III	
TgPgBr 9	I	I	III	I	I	III	
TgPgBr 10	u-1	I	III	III	I	III	
TgPgBr 13	I	I	III	I	u-1	III	
TgPgBr 16	I	I	III	I	I	III	

*u-1* atypical alleles, *ND* not determined

### Genetic characterization by multilocus PCR sequencing

A total of 1,604 positions were used in the alignment to calculate the evolutionary rate among the concatenated sequences of the samples TgCsBr01, TgCsBr02, and TgCsBr03, which was indicated through Tajima’s relative rate test. There were 1,461 identical sites and no divergent sites between the three samples. The null hypothesis of equal rates among the lineages was rejected (*P* < 0.05), and one of the three samples (TgCsBr01) was considered to be from a different lineage.

The samples differed from the 10 strains of *T. gondii* and the apicoplast genome, presenting a mean of 120.6 DNA polymorphisms (5.2 %), including insertion, deletion, transition, and transversion, distributed over the different genetic markers (Table 2).

**Table 2:** Genotyping by PCR-RFLP and number of polymorphisms at six genetic loci detected by PCR sequencing

		No. of polymorphisms detected by sequencing					
Isolate	Genotype PCR-RFLP	Indel	Ts	Tv	Total	Sequence with the highest-scoring segment pairs in ToxoDB	Identity (%); Expected value
Marker SAG1 (225 bp) – Chromosome VIII Coding function: Surface antigen gene							
TgCsBr01	I	0	0	0	0 (0.0 %)	TgUgCh83 (EF534734.1)	100; 4e-113
TgCsBr02	I	0	0	0	0 (0.0 %)	TgUgCh83 (EF534734.1)	100; 4e-113
TgCsBr03	I	0	0	0	0 (0.0 %)	TgUgCh83 (EF534734.1)	100; 4e-113
Marker SAG2 (385 bp) – Chromosome VIII Coding function: Surface antigen gene							
TgCsBr01	I	1	2	3	6 (1.5 %)	TgCkNg1 (EU650330.1)	99; 0.0
TgCsBr02	I	1	2	3	6 (1.5 %)	TgCkNg1 (EU650330.1)	99; 0.0
TgCsBr03	I	7	4	3	14 (3.6 %)	TgCkNg1 (EU650330.1)	97; 0.0
Marker SAG3 (115 bp) – Chromosome XII Coding function: Surface antigen gene							
TgCsBr01	III	0	0	0	0 (0.0 %)	Tg strain CTG (JX218227.1)	100; 3e-52
TgCsBr02	III	1	1	1	3 (2.6 %)	Tg strain CTG (JX218227.1)	99:2e-49
TgCsBr03	III	2	0	0	2 (1.7 %)	Tg strain CTG (JX218227.1)	98:5e-44
Marker c22-8 (485 bp) – Chromosome Ib Coding function: unknown “conserved hypothetical protein”							
TgCsBr01	I	47	64	72	183 (37.7 %)	TgCatBr5 (EU258488.1)	90; 1e-94
TgCsBr02	III	2	3	0	5 (1.0 %)	Tg PTG (EU258476.1)	100; 0.0
TgCsBr03	III	3	1	1	5 (1.0 %)	Tg PTG (EU258476.1)	98; 0.0
Marker PK1 (660 bp) – Chromosome VI Coding function: Protein serine/threonine kinase gene							
TgCsBr01	III	5	0	1	6 (0.9 %)	TgCkNg1 (EU650328.1)	99; 0.0
TgCsBr02	I	5	0	1	6 (0.9 %)	TgCkNg1 (EU650328.1)	99; 0.0
TgCsBr03	I	0	0	1	1 (0.1 %)	TgCkNg1 (EU650328.1)	99; 0.0
Marker Apico^a^ (461 bp) – Apicoplast chromosome							
TgCsBr01	III	1	0	2	3 (0.6 %)	*T. gondii* Apicoplast, comp. genome (U87145.2)	99; 0.0
TgCsBr02	III	42	27	52	121 (26.2 %)	*T. gondii* Apicoplast, comp. genome (U87145.2)	95; 5e-30
TgCsBr03	III	1	0	0	1 (0.2 %)	*T. gondii* Apicoplast, comp. genome (U87145.2)	99; 0.0
Total of polymorphisms at six different genetic loci detected by PCR sequencing of *T. gondii* isolates^b^							
Total of polymorphisms (%)							
Isolate	Genotype PCR-RFLP	Indel	Ts	Tv	Total	Tajima’s relative rate test^c^	Tajima’s *D* neutrality test^d^
TgCsBr01	Atypical	54	66	78	198 (8.5 %)	u = 137	
TgCsBr02	Atypical	51	33	57	141 (6.0 %)	u = 2	
TgCsBr03	Atypical	13	5	5	23 (1.0 %)	u = 4	
average between samples		39.3	34.6	46.6	120.6 (5.2 %)	*P* = 0.00000	*D* = 0.372232

^a^The sequences were aligned with the *T. gondii* apicoplast complete genome

^b^The number of insertions and deletions (Indel), transitions (Ts) and transversions (Tv) were calculated comparing the sequence of each isolate with the pattern obtained from GT1, ME49, VEG, TgCATBr5, TgCATBr9, FOU, RUB, VAND, p89, MAS, TgPgBr06, TgPgBr07, TgPgBr08, TgPgBr09, TgPgBr10, TgPgBr11, TgPgBr12, TgPgBr13, TgPgBr14, TgPgBr15, TgPgBr16, 54, 124 and 127 reference strains. The size of each amplicon means the number of base pairs that matched in all samples after the multiple alignment

^c^The equality of evolutionary rates between the sequences TgCsBr01, TgCsBr02 and TgCsBr03. “u” means unique differences in each sequence. All positions containing gaps and missing data were eliminated. There were a total of 1604 positions with 1461 identical sites in all three sequences and 0 divergent sites between all three sequences. A *P*-value less than 0.05 is often used to reject the null hypothesis of equal rates between lineages

^d^The analysis involved 27 multi-locus nucleotide sequences (GT1, ME49, VEG, TgCATBr5, TgCATBr9, FOU, RUB, VAND, p89, MAS, TgPgBr06, TgPgBr07, TgPgBr08, TgPgBr09, TgPgBr10, TgPgBr11, TgPgBr12, TgPgBr13, TgPgBr14, TgPgBr15, TgPgBr16, 54, 124, 127, TgCsBr01, TgCsBr02, TgCsBr03). All positions containing gaps and missing data were eliminated. There were a total of 1870 bases aligned with 388 segregating sites. A negative Tajima’s *D* indicates an excess of low-frequency polymorphisms. Evolutionary analyses were conducted in MEGA6

Through Tajima’s *D* test, which measures the distribution of allele frequency based on the data of nucleotide sequences, 1,870 bases could be aligned, showing 388 segregating sites. The analysis showed a positive result (0.372), which was indicative of a low number of low-frequency polymorphism among all the strains of *T. gondii* used in this analysis.

### Phylogenetic analysis

**Fig. 1:**
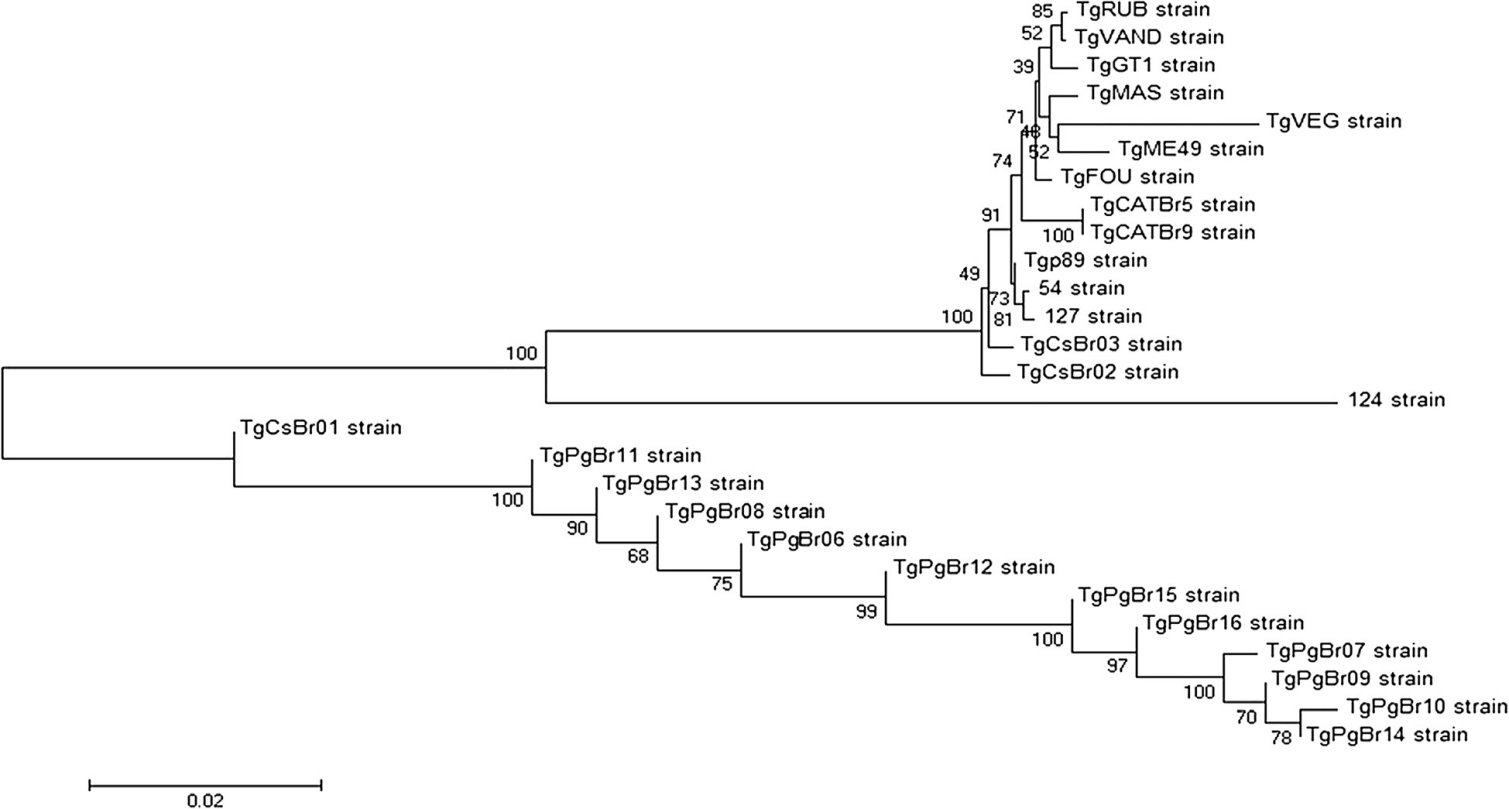
Phylogram of *Toxoplasma gondii* strains as determined by sequence analysis of the five genomic markers

The phylogenetic analysis of the DNA sequences by the neighbor-joining method demonstrated that TgCsBr02 and TgCsBr03 were grouped in a group distinct from TgCsBr01. TgCsBr02 and TgCsBr03 were considered a sibling group, defined by an apomorphy of 99 % of the bootstrap replicates. TgCsBr01 presented the largest branch length, which was proportional to the amount of polymorphisms. The isolates TgCsBr02 and TgCsBr03 were the closest to the reference strains GT1 (type I), ME49 (type II), and VEG strain (type III), but were grouped in a distinct group with 15 % of the bootstrap replicates (Fig. 1).

## Discussion

This was the first study that identified and genetically characterized *T. gondii* from *C. subspinosus*. None of the samples were classified as a clonal genotype of type I, II, or III (Table 1) or as a main Brazilian clonal genotype (BRI, BRII, BrIII, and BRIV) defined by Dubey *et al.* [16] and Pena *et al.* [2]. The samples were also genetically distinct from genotypes of *T. gondii* previously isolated from pigs and sheep of the same geographic region of Brazil (southern Bahia), as described by Bezerra *et al.* [3] and Maciel *et al.* [4] (Table 1). Moreover, the samples did not combine with any genotype deposited in ToxoDB.

However, the phylogram determined by the analysis of the chromosomal genetic marker sequence revealed that the isolates TgCsBr02 and TgCsBr03 were closer to the ovine strains of the same area of study (#54 and #127). TgCsBr01 shared a monophyletic group in 99 % of the replicates with swine isolates from the same geographical region (TgPgBr06, TgPgBr07, TgPgBr08, TgPgBr09, TgPgBr10, TgPgBr11, TgPgBr12, TgPgBr13, TgPgBr14, TgPgBr15, and TgPgBr16), which suggests that it may belong to a close lineage that inhabits this region. However, the genetic characterization of the parasite carried out by means of multilocus PCR-RFLP and DNA sequencing techniques indicated a high genetic diversity of the parasite in the region (Tables 1 and 2; Fig. 1).

The genetic characterization by PCR-RFLP revealed two genotypes in the three strains. Nevertheless, it was verified that the isolates of *C. subspinosus* possessed many of the same alleles present in other Brazilian genotypes, although the alleles had distinct segregation among the loci examined, thereby characterizing it as a new genotype. The multilocus PCR sequencing indicated that the three isolates from the *C. subspinosus* samples were distinct, and the genetic variability between the samples was enough to classify them into different genotypes in the phylogenetic analysis. This was indicated by Tajima’s relative rate test, which calculated the equality of evolutionary rates between the concatenated sequences generated by multilocus PCR sequencing (Fig. 1).

The most polymorphic genetic markers were c22-8 and Apico, with alignments presenting 90 and 95 % identity with the TgCatBr5 and apicoplast genomes, respectively (Table 2). Despite this high degree of polymorphism, the samples were grouped in the clonal genotype type III by the PCR-RFLP technique (Table 1). These regions may be considered effective to distinguish isolates of clonal types I, II, and III, though this complicates the grouping of Brazilian isolates.

Few studies have genetically characterized *T. gondii* in wild mammals, and most research in Brazil is related to serological techniques in these animals [5, 6, 17, 18]. Most isolates of *T. gondii* genotyped in Brazil are from domestic animals, including chickens, cats, dogs, sheep, goats and pigs. The studies that have genetically characterized isolates of *T. gondii* from wild animals have also reported a high genetic diversity and have contributed to the elucidation of the Brazilian genotype network [19–21]. Silva *et al.* [6], upon detecting *T. gondii* in armadillos in Brazil, claimed that these wild animals can be a source of transmission of the parasite to humans, mainly due to the rural people’s habit of eating the meat of these animals.

## Conclusions

Little is known about the genetic variability of isolates of *T. gondii* in wild mammals in Brazil. Knowing the genetic similarities or differences of *T. gondii* between different animal populations is necessary to understand the transmission of the parasite.
